# Central Pancreatectomy with Roux-en-Y Pancreaticojejunal Anastomosis—Report of Two Cases

**DOI:** 10.1055/s-0040-1718699

**Published:** 2020-12-14

**Authors:** Roza Panagis Moureletou, Dimitrios Kalliouris, Konstantinos Manesis, Sotirios Theodoroleas, Angeliki Bistaraki, George Boubousis, Efstathios Nikou

**Affiliations:** 1 2nd Department of General Surgery, 417 Army Share Fund Hospital, Athens, Greece; 2 Surgery Department, 417 Army Share Fund Hospital, Athens, Greece

**Keywords:** central pancreatectomy, pancreaticojejunal anastomosis, pNET

## Abstract

**Background** Central pancreatectomy (CP), a partial resection of the pancreas, is indicated for the excision of neuroendocrine tumors (NETs) of the pancreas, when located at the neck or the proximal body. Specifically, CP is preferable in functional NET and in nonfunctional sized 1 to 2 cm or/with proliferation marker Ki67 < 20% (Grade I/II). Postoperative leakage from the remaining pancreas constitutes the most frequent complication of CP (up to 63%). The aim of our study was to share the experience of our center in CP for NET, with pancreaticojejunal anastomosis.

**Methods** In 1 year, we performed CP in two patients, following the aforementioned criteria. They presented with tumor of the body of the pancreas, which was found in random check with computed tomography, with negative hormonal blood tests and they underwent magnetic resonance imaging and endoscopic ultrasound/fine-needle biopsy/pathological examination.

**Results** The patients underwent CP with Roux-en-Y pancreaticojejunal anastomosis of the distal pancreatic stump and jejunal patch of the proximal pancreatic stump. Histological exam revealed NET sized 2.8 cm and 1.45 cm, Grade I and II, respectively. Postoperatively both patients developed small pancreatic leakage, which did not affect their physical condition and stopped after 20 and 30 days. No one needed pancreatic enzymes supplements or developed new-onset diabetes mellitus.

**Conclusion** CP provided adequate, functional remaining pancreatic tissue in both patients. Small leakages were treated conservatively and retreated without septic complications. As a result, CP might be considered as safe and effective technique for pancreatic neck/proximal body NET.

The uprising indications for abdomen computed tomography (CT) scan have led to increased diagnosis of low or intermediate malignancy tumors in the past few years. 1 Neuroendocrine tumors of the pancreas (pNETs) constitute as a rare, heterogeneous group of pancreatic neoplasms arising from the neuroendocrine system of the gut. It is estimated that less than 3% of pancreatic tumors prove to be pNETs. They may coexist with pituitary adenoma and parathyroid hyperplasia within the multiple endocrine neoplasia type 1. pNETs are divided into two groups, functional (F-pNETs) and nonfunctional (NF-pNETs) tumors and they appear to have a wide prognostic range, because they may have benign, uncertain, or malignant behavior. 2 In 2010, World Health Organization (WHO) offered a histological classification of pNETs, which were divided based on the Ki67% index into three groups: Grade I when Ki67≤2%, Grade II when Ki67 = 3–20%, and Grade III when Ki67 > 20%, and this classification was clinical validated to successfully predict metastases or recurrences. 3

Therapeutically, pancreatoduodenectomy (PD) and distal pancreatectomy (DP) with or without preserving the spleen have been used for the resection of benign or intermediate malignancy tumors, such as pNETs. However, these techniques resulted in the resection of a large portion of healthy pancreatic tissue, increasing the risk of pancreatic endocrine and exocrine disfunction. Large population studies revealed that the incidence of new-onset diabetes mellitus (NODM) and exocrine pancreatic insufficiency after PG was up to 22.2 and 49.1%, respectively. 4 Also NODM occurrence ranges in 14–30.5% after DP. 5 6

Furthermore, enucleation of benign lesion such as insulinomas has also been performed. However, enucleation of tumors located at the pancreatic body is not always feasible, because the tumors often infiltrate to the main pancreatic duct. As a result, an alternative technique has been proposed, called central pancreatectomy (CP), for the resection of solitary, small tumors located at the pancreatic neck or proximal body, which do not need extended lymphatic clearance, preserving as much healthy pancreatic tissue as possible. 7

CP is a parenchyma-sparing segmental resection where the cephalic stump is sutured or anastomosed to a Roux-en-Y jejunal loop—the distal stump is anastomosed to the aforementioned loop or to the stomach. It was first performed in 1957 by Guillemin and Bessot for a patient with pancreatitis 8 later it was performed by Letton and Wilson for severe traumatic injury of the pancreatic body 9 and afterward it had limited use in trauma and benign conditions. CP had limited use until 1998 that became apparent that CP had a place in pancreatic surgery for benign or low-grade malignant tumors. 10 In 2008, a study regarding a single-center experience of 50 cases with low-grade malignant neoplasms concluded that CP leads to effective preservation of both cephalic and distal pancreatic remnants without a significant increase in postoperative morbidity compared with conventional pancreatectomy. 11 A study in 2010 revealed that CP had lower rate of new-onset, lower rate of worsening diabetes and decreased insulin requirements, compared to DP. 12

Laparoscopic CP has been previously performed 13 and CP without anastomosis has also been reported, as an effective procedure with lower morbidity and reduced length of hospital stay, compared with patients undergoing CP with an anastomosis. 14

It is of note that CP has been successfully performed for the treatment of various conditions. Specifically, CP is indicated for the resection of intraductal papillary mucinous neoplasm (IPMN), mucinous cystic neoplasm, serous cystadenomas, solid pseudopapillary neoplasms and nonneoplastic cysts not suitable for enucleation, isolated metastases to the pancreas, focal chronic pancreatitis with Wirsung's duct stenosis, and pancreatic trauma. 15 16 An exception to these indications could be the IPMN of the main duct, due to the difficulty of preoperative and intraoperative diagnosis of definite negative margins, thus increasing the risk for recurrence. 7

In the recent consensus guidelines update for the management of patients with pNETs, surgical resection for NF-pNET is preserved for patients with tumors > 2cm and tumors < 2 cm Grade II (based on proliferation index Ki67 <20%). Surgical intervention could be enucleation or local resection, while more extended resections, such as PD and DP, are reserved for selected cases. 17

The objective of our study was to share the experience of our center in open CP with pancreaticojejunal anastomosis (PJA) for pancreatic neck/proximal body NF NETs.

## Materials and Methods

**Fig. 1:**
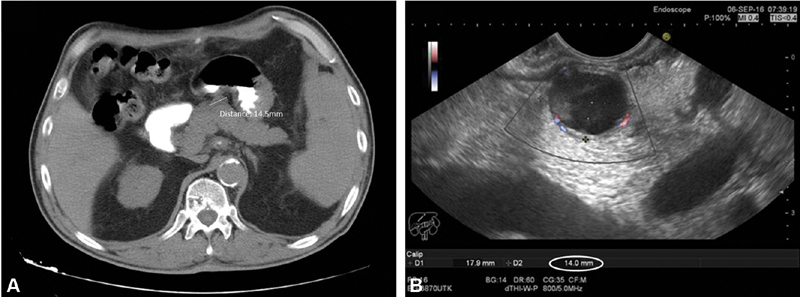
( **A** ) computed tomography and ( **B** ) endoscopic ultrasound of patient 1 showing the pancreatic lesion.

In 1 year, two male patients were admitted to our department with asymptomatic pancreatic lesion, which was found incidentally on CT scan. Patient 1 was 77 years of age and his medical history revealed arterial hypertension, hyperuricemia, diabetes mellitus II (diagnosed 4 years ago), and carotid disease ( Table 1 ). He was tested for gastrin, chromogranin A, adrenocorticotropic hormone (ACTH), and growth hormone (GH) secretion and was found negative. Subsequently, magnetic resonance imaging (MRI) and endoscopic ultrasound/fine-needle biopsy/pathological examination were performed to determine the location, size, and proliferation index Ki67 of the lesion. MRI revealed mass at the proximal pancreatic body of 1.45 cm in patient 1, but the EUS revealed mass with size 1.7 × 1.4 cm ( Fig. 1 ). Histopathological examination of biopsy taken from the EUS of the patient 1 revealed pNET with Ki67 3 to 20% (Grade II).

**Table 1:** Clinical and postoperative data

	Gender	Age	Medical history	Type of surgery—duration (min)	Duration of hospital stay (d)	Drainage amylase levels mean (min—max) IU/L	Duration of leakage (d)	Grade of fistulae a
Patient 1	M	77	AH, HU, DMII, carotid disease	CP with PJA (360)	18 (1st) 6 (2nd)	16,072 (1,568–38,823)	30	B
Patient 2	M	72	AF, sleep apnea, renal impairment	CP with PJA (445)	15	11869 (3,858–34,235)	20	Biochemical leak (former A)

Abbreviations: AF, atrial fibrillation; AH, arterial hypertension; CP, central pancreatectomy; DM II, diabetes mellitus II; HU, hyperuricemia; PJA, pancreaticojejunal anastomosis.

a Based on 2016 revised criteria of the International Study Group on Pancreatic Fistula.

**Fig. 2:**
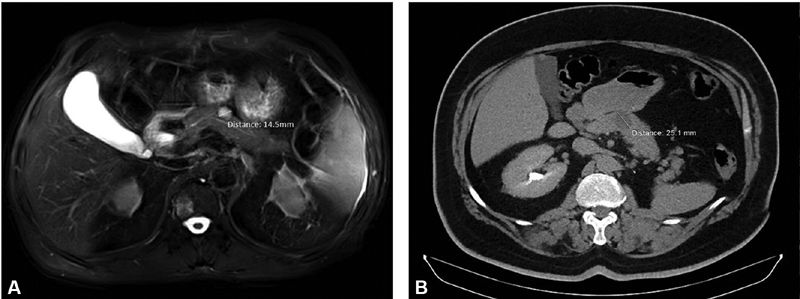
Magnetic resonance imaging showing the pancreatic lesion of ( **A** ) patient 1 and ( **B** ) patient 2.

Patient 2 was 72 years of age and his medical history included atrial fibrillation, sleep apnea, and renal impairment, without the need for dialysis ( Table 1 ). His blood sample turned out to be negative for gastrin, chromogranin A, ACTH and GH secretion, and the patient also had normal bilirubin levels. The MRI of patient 2 revealed hypervascular solid mass of proximal pancreatic body of 2.51 cm, compatible with pNET, without pathological lymph nodes or apparent distant metastasis ( Fig. 2 ). Since his lesion exerted the limit of 2 cm and based on the MRI characteristics, it was decided to proceed with CP rather than enucleation, due to the high risk of injuring the Wirsung's duct. 18

As a result, both our patients exhibited NF pNET sized > 2cm or with proliferation marker Ki67 < 20%; thus, they were fulfilling the criteria for segmental pancreatectomy. 17 Informed consent of the patients was obtained following a detailed explanation of the procedure and the associated risks.

## Results

**Fig. 3:**
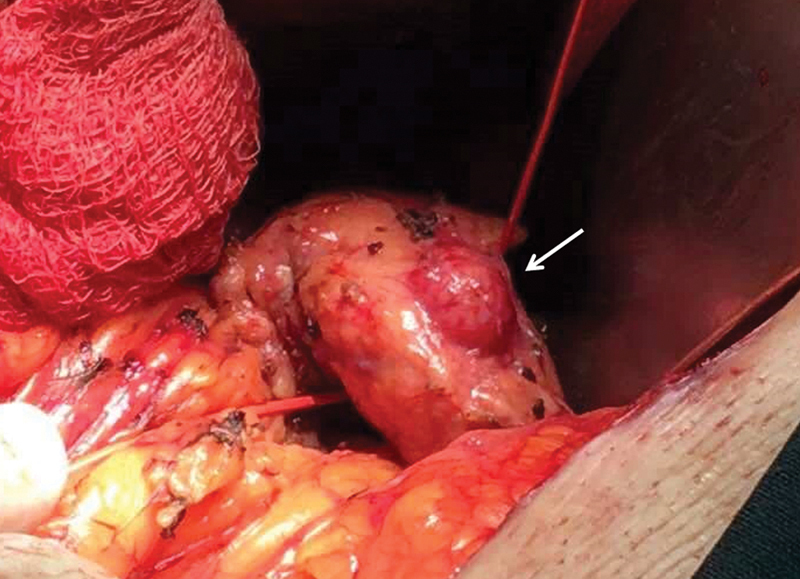
During surgery, the posterior wall of pancreatic body is getting detached from the superior mesentery vessels. Arrow indicates the lesion of patient 2.

**Fig. 4:**
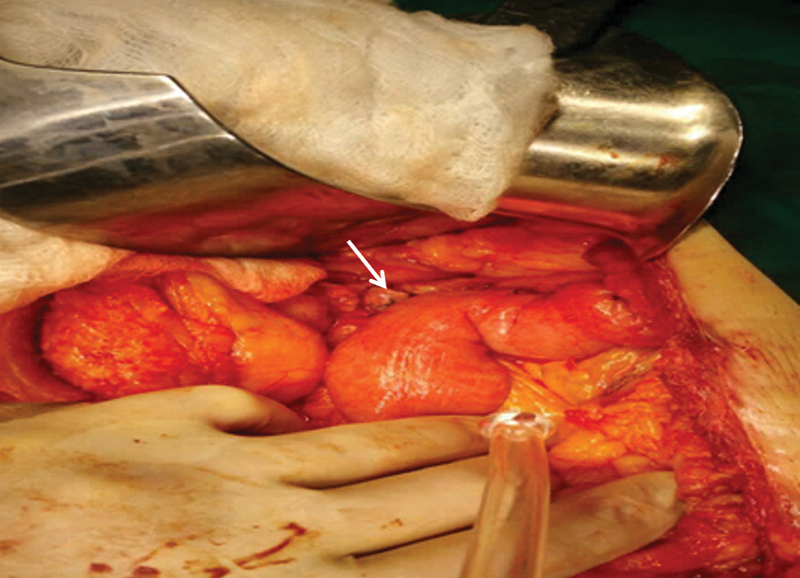
Creation of pancreaticojejunal anastomosis with jejunal patch (as indicated by the arrow).

Both our patients underwent CP with distal PJA with Roux-en-Y reconstruction. Regarding the surgical technique, after Kocher maneuver, the posterior wall of pancreatic body was detached from the superior mesentery vessels ( Fig. 3 ) and the proximal body was resected, yielding 1 cm free of the lesion. The splenic vessels were retained. The proximal remaining pancreas was primarily closed with hemostatic sutures (horizontal mattress). Afterward, a jejunal loop was used for the creation of a handmade end-to-side PJA with the distal end of the remaining pancreas in two layers of sutures. At the same jejunal loop, just a few cm further of the anastomosis, the head and neck of the pancreas were approximated using complementary sutures, as a jejunal patch ( Fig. 4 ). In both the patients, drain was placed near the PJA.

Pathology examination of patient 1 revealed pNET, sized 1.45 cm, Grade II. Histopathology examination of the patient's 2 specimen confirmed the diagnosis of pNET Grade I with lesion size 2.8 cm. In both the specimens, the margins were free.

Early postoperatively both patients exhibited signs of pancreatic fistula (PF) but reoperation or interventional radiologic procedures were not required in any of them. The fistulae of patients 1 and 2 were classified based on the 2016 revised criteria of the International Study Group on Pancreatic Fistula as Grade B (persistent drainage >3 weeks) and biochemical leak (former Grade A), respectively 19 20 ( Table 1 ).

Specifically, patient 1 from the 1st postoperative day (POD) exhibited elevated amylase levels from the abdominal drain (mean amylase 16,072 IU/L, ranged between 1,568 and 38,823 IU/L), with output less than 200 mL/day and leukocytosis until the 10th POD, though he did not present with fever or hemodynamic instability. Patient 1 was discharged on the 16th POD with the drain in place. The patient came back for follow-up on the 24th POD with mild abdominal discomfort and leukocytosis. CT scan was performed, which identified small intraperitoneal fluid collection near the PJA (∼100 mL). Readministration to the hospital was decided for broad-spectrum antibiotic (ciprofloxacin and metronidazole) treatment. The patient had significant clinical improvement, and the drainage gradually dropped to 20 mL/day. As a result, the drain was removed 6 days later, the patient discharged on the same day, and he was in excellent physical condition during the next follow-up visit.

On the other hand, patient 2 presented on the 1st POD with a biochemical leak with amylase more than three time times of the upper limit of the normal serum amylase (mean amylase from drainage 11,869 IU/L, ranged between 3,858 and 34,235 IU/L), with output equal or less than 100 mL/day, without septic complications (fever, leukocytosis, etc.). Otherwise his postoperative course was uncomplicated and he was discharged on the 13th POD. He came back a week later (on the 20th POD) for clinical examination, was found in good clinical condition and had his abdominal drain removed, since the daily output was less than 20 mL/day.

After discharge, both patients were advised to get a thorough evaluation from an oncologist. At 6 months follow-up, none of them was needed complementary administration of pancreatic enzymes, none of them developed NODM II, and none of them had a recurrence of the disease.

## Discussion

CP compared with PD and DP showed significantly reduced mortality and lower rates of postoperative deaths, making CP preferable for benign or low-grade malignant tumors. 21 22 As far as the function of the remaining pancreatic tissue is concerned, in a median follow-up of 36 months after CP, the rates of new-onset exocrine and endocrine insufficiency were 6 and 2%, respectively. 23

In our study, none of our patients reported signs/symptoms of exocrine dysfunction, such as steatorrhea, flatulence, weight loss, and/or other signs of unjustified malnutrition and none of them reported NODM. It is worth mentioning that patient 1 already had diabetes mellitus II, diagnosed several years prior to surgery and was under treatment with vildagliptin, metformin/glimepiride. After surgery, he needed insulin to maintain glucose control, though his glycemic control even before surgery was poor, as indicated by high (ranged between 7.5 and 8.5%.) glycated hemoglobin A1c.

Regarding the surgical technique, we prefer pancreatectomy with PJA to pancreatogastric anastomosis. Previous studies have shown that pancreatogastric anastomosis was associated with higher risk of abdominal postoperative complications, such as abdominal collections and PF. 24 On the other hand, a large meta-analysis concluded that there were no differences regarding the rate of clinically significant PF, postoperative hemorrhage, or delayed gastric emptying. 25

In our study, none of our patients exhibit postoperative hemorrhage, but both presented with PF; the first had only biochemical leakage and the other one developed Grade B PF, without septic complications. In many studies, PF has been described as the most common complication after CP, with an incidence variation up to 63%. 23 24 One explanation for this high prevalence of post-CP formation of PF lays to the fact that CP has two points that might leak, the proximal head stump, which is “patched” to the jejunum, and the distal one, which is anastomosed to the jejunal loop. Furthermore, the anastomosis is performed on a soft pancreas usually with a normal diameter main pancreatic duct, because of the benign or low-grade malignant tumors that constitute indications for CP. 24

With respect to the limitations issued by our small sample, we might conclude that CP as a parenchyma-sparing technique could provide adequate, functional remaining pancreatic tissue, because none of our patients developed NODM or needed pancreatic enzymes supplements. Furthermore, small pancreatic leakage (Grade A or B) could be treated conservatively and diminished automatically without septic complications. In conclusion, CP with Roux-en-Y PJA and jejunal patch to the proximal pancreatic stump might be considered as safe and effective technique for low or intermediate malignancy tumors.
